# Global trends in polycystic ovarian syndrome over 30 years: an age-period-cohort study of 204 countries and territories (1990–2021)

**DOI:** 10.1186/s41043-025-01002-1

**Published:** 2025-07-08

**Authors:** Lanxiang Lin, Wenhui Li, Baozhu Xu, Yuefeng Li, Jin Li, Xiangjun Kong, Xin Du

**Affiliations:** 1 Health Management Center, Xiang’an Hospital of Xiamen University, School of Medicine, Xiamen University, Xiamen, 361102 China; 2 Intravenous Drug Dispensing Centre, Xiang’an Hospital of Xiamen University, School of Medicine, Xiamen University, Xiamen, 361102 China; 3 Department of Ultrasound, Xiang’an Hospital of Xiamen University, School of Medicine, Xiamen University, Xiamen, 361102 China

**Keywords:** Polycystic ovary syndrome, Age-period-cohort, Incidence, Global burden of disease study, Global health

## Abstract

**Purpose:**

To characterize global temporal trends in the incidence of polycystic ovary syndrome (PCOS) from 1990 to 2021 by applying an age–period–cohort (APC) analytical framework across 204 countries and territories, and to assess how these incidence patterns vary according to socio-demographic context.

**Methods:**

This study analyzed PCOS incidence trends from 1990 to 2021 across 204 countries using an APC analysis. Data were sourced from the Global Burden of Disease Study 2021, focusing on crude and age-standardized incidence rates, disaggregated by region and socio-demographic index (SDI).

**Results:**

The global incidence of PCOS increased from 1.48 million cases in 1990 to 2.3 million in 2021. The age-standardized incidence rate in 2021 was 63.26 per 100,000 population (95% CI: 45.41 to 87.28). High SDI regions recorded the highest incidence rates, with a rate of 90.13 per 100,000 (95% CI: 66.94 to 122.36). In contrast, low SDI regions had the lowest incidence rates at 36.8 per 100,000 (95% CI: 25.83 to 52.19). The APC model revealed a global net drift of 0.18 (95% CI: -0.02 to 0.37), with significant age and period effects, particularly among younger age groups.

**Conclusion:**

This APC analysis reveals marked increases in global PCOS incidence driven by adolescent age groups, mid-2000s period effects, and recent birth cohorts. Findings underscore the need for age- and cohort-targeted interventions and harmonized diagnostics to address the growing PCOS burden.

**Supplementary Information:**

The online version contains supplementary material available at 10.1186/s41043-025-01002-1.

## Introduction

Polycystic ovary syndrome (PCOS) is one of the most common endocrine disorders among women of reproductive age, affecting an estimated 5–20% of women globally, depending on the diagnostic criteria used [1]. The syndrome is characterized by a combination of hyperandrogenism, ovulatory dysfunction, and polycystic ovaries, and it is often associated with metabolic abnormalities such as insulin resistance, obesity, and dyslipidemia [2]. Over the past three decades, the prevalence of PCOS has increased markedly, driven primarily by the global obesity epidemic. Notably, the worldwide age-standardized prevalence of obesity nearly tripled between 1990 and 2021, with projections indicating that over 3.8 billion adults will be affected by 2050 [3]. This escalation in obesity rates parallels a significant rise in associated metabolic disorders, such as type 2 diabetes, which affects approximately 10% of adults worldwide [4] and nonalcoholic fatty liver disease (NAFLD), now recognized as the most prevalent liver disorder, affecting up to 30% of the global population [5]. Given that obesity and its related metabolic complications are major risk factors for PCOS, these trends provide critical context for understanding the syndrome’s growing incidence. Alongside obesity, additional factors such as sedentary lifestyles and shifts in reproductive behaviors further contribute to the rising prevalence of PCOS [6]. Consequently, PCOS has emerged as a pressing public health challenge, with profound implications for women’s reproductive health, fertility, and overall quality of life.

In addition to clinical and metabolic features, ultrasound imaging plays a critical role in the diagnosis and management of PCOS. Ultrasonography, particularly transvaginal ultrasound, is instrumental in identifying polycystic ovarian morphology [7] which is defined by the presence of 12 or more follicles in each ovary measuring 2–9 mm in diameter, or increased ovarian volume exceeding 10 cm³ [8]. Recent advances in ultrasound technology have enhanced the ability to detect subtle ovarian changes, thereby improving the diagnostic accuracy for PCOS. For instance, three-dimensional (3D) ultrasound offers more precise volumetric assessment of the ovaries and follicle counts, which has refined the criteria for diagnosing PCOS and distinguishing it from other ovarian disorders [9]. Studies have shown that the incorporation of advanced ultrasound techniques not only aids in the early detection of PCOS but also in monitoring the response to treatment, particularly in managing infertility associated with the syndrome [10]. These imaging advancements underscore the need for a multidisciplinary approach in managing PCOS, integrating both clinical and imaging data to optimize patient outcomes.

Despite the significant role of ultrasonography in PCOS management, most existing research has concentrated on the prevalence of PCOS rather than its incidence [11, 12] leaving a critical gap in understanding the dynamics of new cases over time. Prevalence studies provide valuable insights into the overall burden of the disease but fail to capture the rate at which new cases are emerging. Incidence data are essential for understanding the temporal trends in PCOS, including the effects of changing population risk factors such as age, socioeconomic status, and lifestyle behaviors. Furthermore, incidence analysis can inform the effectiveness of public health interventions aimed at reducing the occurrence of PCOS and its associated complications.

Despite the importance of incidence data, research on PCOS incidence has been limited, often restricted to specific regions or population subgroups [13] which hinders a comprehensive understanding of global trends. Additionally, longitudinal studies covering multiple decades are scarce, making it difficult to assess how PCOS incidence has evolved in response to global changes in factors such as urbanization, dietary patterns, and healthcare access. This lack of longitudinal, global data limits the ability to develop effective, targeted public health strategies to address the rising burden of PCOS.

In response to this gap in knowledge, the present study seeks to investigate the trends in PCOS incidence using an age-period-cohort (APC) analysis across 204 countries and territories from 1990 to 2021. The APC model is particularly well-suited for disentangling the effects of age, period, and cohort influences, providing detailed insights into the factors driving changes in PCOS incidence over time [14]. By analyzing three decades of global data, this study aims to identify key patterns and shifts in the incidence of PCOS, thereby shedding light on the demographic, economic, and lifestyle factors that have contributed to the observed trends.

The findings of this study are expected to have significant implications for public health policy, particularly in designing interventions to curb the rising incidence of PCOS. By filling a crucial gap in the understanding of PCOS incidence trends, this research will contribute to the broader body of knowledge on PCOS and provide a foundation for future studies aimed at mitigating the global impact of this increasingly common condition.

## Materials and methods

### Data source

This study utilizes incidence data for PCOS derived from the GBD 2021 database. The GBD dataset integrates data from a wide array of global sources, including scientific literature, epidemiological surveys, disease registries, clinical informatics, and government reports [15]. The GBD methodology involves adjusting data for biases through meta-regression techniques, with network meta-regression (MR-BRT) employed to correct for measurement inconsistencies. Incidence data for PCOS are made available for 204 countries and territories, spanning from 1990 to 2021, and categorized by sex, region, age, year, and socio-demographic index (SDI) [16]. To account for discrepancies in measurement techniques across regions, hierarchical modeling approaches are used, which include the DisMod-MR 2.1 and spatiotemporal Gaussian process regression (ST-GPR) models. These methods facilitate the generation of internally consistent estimates of PCOS incidence across different age groups and locations [16, 17].

The study focused on PCOS incidence data as defined by the NIH and American College of Obstetricians and Gynecologists (ACOG), where PCOS is diagnosed based on chronic anovulation and hyperandrogenism, confirmed by clinical findings and hormone levels, excluding secondary causes [18]. We analyzed both all-age incidence rates (AAIR) and age-standardized incidence rates (ASIR), which were calculated using the GBD population structure, which adjusts for the global age distribution [15].

### Statistical analysis

#### Analysis of temporal trends in PCOS incidence

The trends in PCOS incidence were analyzed across all age groups, with particular focus on the temporal variations from 1990 to 2021. The data were stratified by age categories, with standard age-group intervals spanning from 10 to 54 years. Incidence rates were reported per 100,000 person-years, both AAIR and ASIR, using the global age structure from the GBD 2021 study [15].

#### Age-period-cohort modeling analysis of incidence data

To assess the effects of age, period, and cohort on the incidence of PCOS, we employed an APC analysis. This method differentiates the contributions of three temporal factors: age effects, which are biological and social changes due to aging; period effects, which arise from external factors such as environmental changes or health interventions; and cohort effects, which reflect unique experiences or exposures of birth cohorts over time [19].

The APC model was applied to data categorized into nine age groups (10–14, 15–19, 20–24, 25–29, 30–34, 35–39, 40–44, 45–49, and 50–54 years) and fourteen overlapping ten-year birth cohorts, spanning from 1936 to 1944 to 2001–2009. The intrinsic estimator method was employed to resolve collinearity issues between the age, period, and cohort variables [20]. The analysis was conducted using two-tailed tests with a significance threshold of *p* < 0.05. Relative risks (RRs) were calculated for each period and cohort, where values greater than 1 indicate an increased risk of PCOS incidence.

Statistical modeling and analyses were performed using R (version 4.4.1), with visualizations generated using R. APC modeling was conducted using the online tool provided by the National Cancer Institute [21, 22] and uncertainty in the estimates was addressed by calculating 95% uncertainty intervals (UIs) through 500 simulation draws.

## Results

### Trends in PCOS incidence rates, 1990–2021

**Fig. 1:**
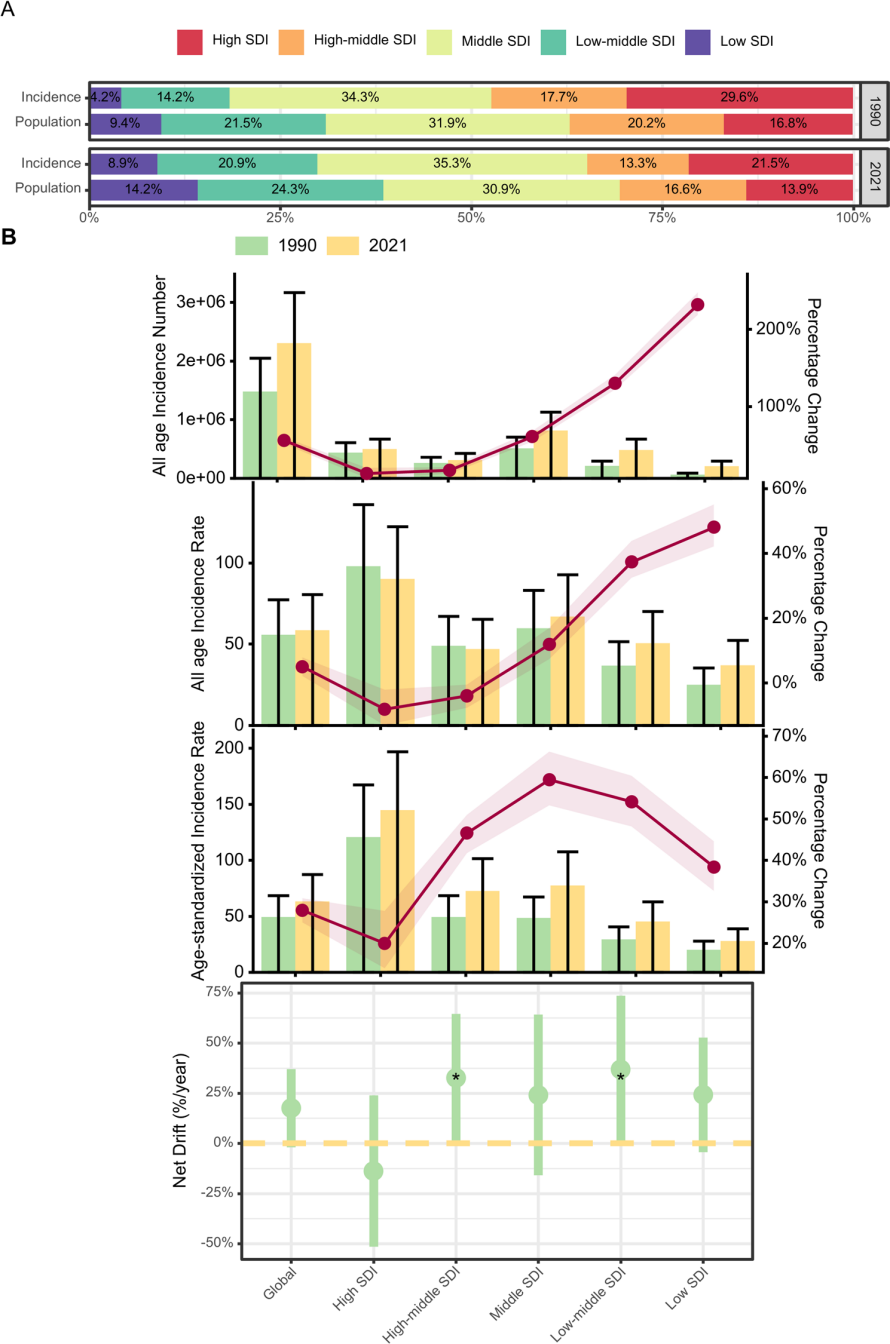
PCOS incidence rates is increasing globally. (**A**) Changes in the proportion of PCOS deaths in five different SDI regions relative to the proportion of the population in each region, 1990 to 2021. (**B**) Trends in PCOS incidence rate across SDI quintiles, 1990 to 2021. PCOS = Polycystic ovary syndrome; SDI = Socio-demographic Index. APC = age-period-cohort

**Fig. 2:**
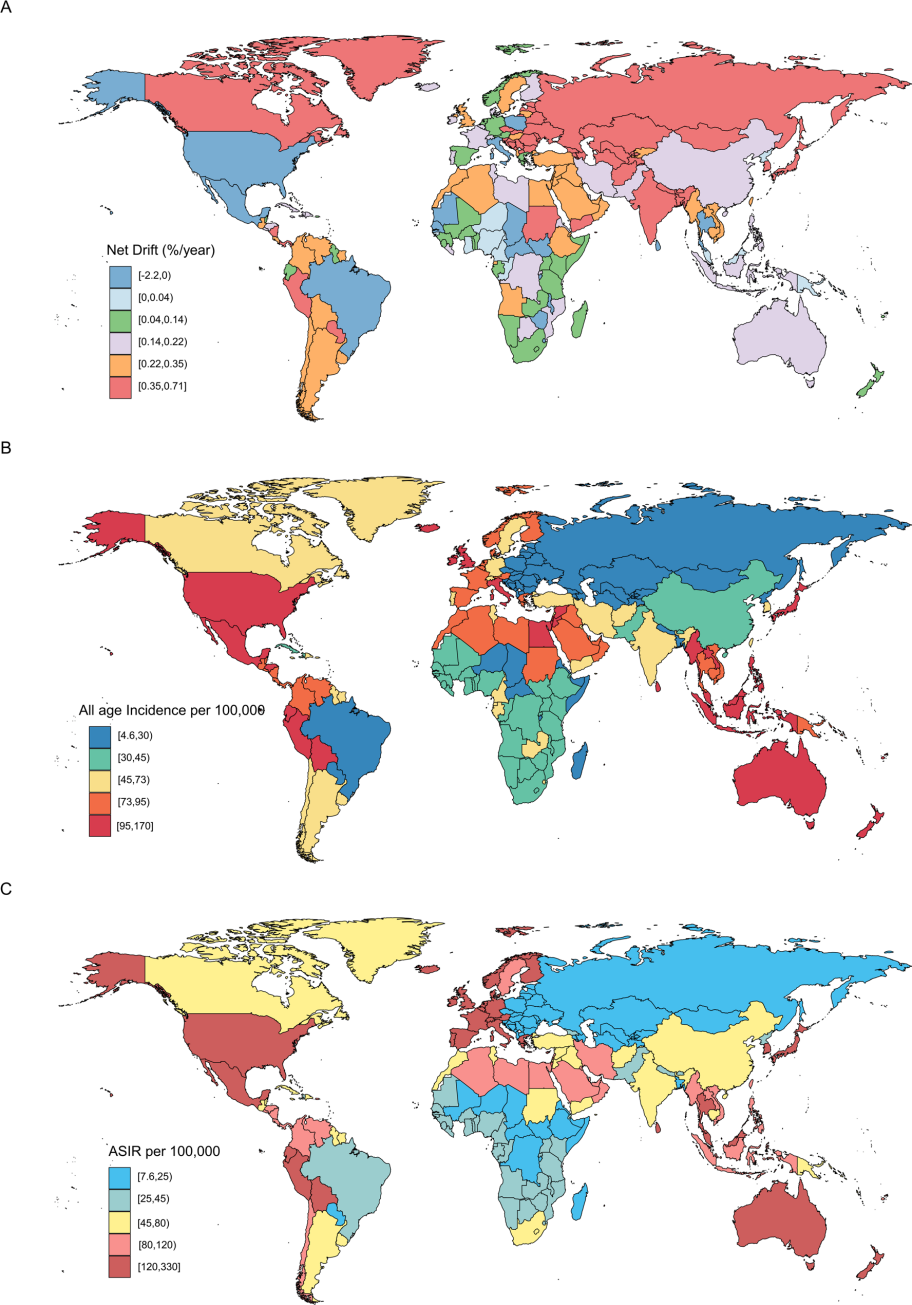
The net drift (**A**), all-age incidence rate (**B**) and ASIR (**C**) in 204 countries and territories. (**A**) World map of net drifts for PCOS incidence rate. Net drift captures components of the trends attributable to calendar time and successive birth cohorts. The global net drift of PCOS incidence rate was 0.18% (95 CI: -0.02, 0.37). (**B**) World map of all-age incidence rate for PCOS in 2021. The global all-age incidence rate was 58.53 (95% CI: 42.12, 80.55) per 100,000 populations. (**C**) World map of ASIR for PCOS in 2021. The global ASIR was 63.26 (95% CI: 45.41, 87.28) per 100,000 populations. PCOS = Polycystic ovary syndrome. ASIR = age-standardized incidence rate. CI = Confidence interval

Table 1; Figs. 1A and B and 2, and Supplementary Table 1 display the trends in PCOS incidence, encompassing population data, total cases, AAIR, ASIR, and net drift over the study period. From 1990 to 2021, the global number of PCOS cases rose from 1,476,225.27 (95% CI: 1,057,983.5 to 2,045,276.94) to 2,301,505.64 (95% CI: 1,655,989.24 to 3,167,177.81). In 2021, the ASIR reached 63.26 (95% CI: 45.41 to 87.28) per 100,000 individuals. The global APC model indicated a net drift of 0.18 (95% CI: -0.02 to 0.37) in PCOS incidence during this period.

**Table 1:** Trends in polycystic ovarian syndrome across SDI quintiles, 1990–2021

location	Population		All-age numbers			All-age incidence			Age-standardized incidence			NetDrift of incidence from APC model, % per year
	Number in 1990	Number in 2021	Number in 1990	Number in 2021	Percent change 1990–2021, %	Rate in 1990	Rate in 2021	Percent change 1990–2021, %	Rate in 1990	Rate in 2021	Percent change 1990–2021, %	
Global	2647880415.27 (2597849840.45, 2701943820.28)	3931961861.58 (3822504340.01, 4049400621.79)	1476225.27 (1057983.5, 2045276.94)	2301505.64 (1655989.24, 3167177.81)	0.56 (0.52, 0.6)	55.75 (39.96, 77.24)	58.53 (42.12, 80.55)	0.05 (0.02, 0.08)	49.45 (35.57, 68.45)	63.26 (45.41, 87.28)	0.28 (0.25, 0.31)	0.18 (-0.02, 0.37)
Low SDI	248967994.55 (242431016.67, 255624488.81)	558274164.12 (534157455.02, 582436040.35)	61881.63 (43355.56, 87726.03)	205458.3 (144223.85, 291379.81)	2.32 (2.19, 2.48)	24.86 (17.41, 35.24)	36.8 (25.83, 52.19)	0.48 (0.42, 0.55)	20.08 (14.45, 28.07)	27.79 (19.81, 38.87)	0.38 (0.33, 0.45)	0.24 (-0.04, 0.53)
Low-middle SDI	570158969.62 (550259425.58, 589169772.61)	955385165.83 (906446878.75, 1005342460.36)	209333.49 (148000.1, 293837.26)	481689.01 (338285.68, 670310.33)	1.3 (1.22, 1.41)	36.71 (25.96, 51.54)	50.42 (35.41, 70.16)	0.37 (0.32, 0.44)	29.32 (21.09, 40.77)	45.2 (31.82, 62.95)	0.54 (0.48, 0.6)	0.37 (0, 0.74)
High SDI	446135451.43 (434991459.83, 457213982.28)	548355501.81 (532972005.14, 564169622.86)	437301.5 (319330.75, 606631.95)	494212.24 (367071.51, 670948.04)	0.13 (0.07, 0.2)	98.02 (71.58, 135.97)	90.13 (66.94, 122.36)	-0.08 (-0.13, -0.02)	120.81 (88.32, 167.22)	144.89 (107.28, 196.81)	0.2 (0.14, 0.28)	-0.14 (-0.51, 0.24)
High-middle SDI	534278941.78 (516799918.11, 552414431.63)	651470409.84 (625545079.47, 678973269.63)	261030.54 (187118.65, 358760.32)	305809.98 (216061.4, 424907.89)	0.17 (0.13, 0.21)	48.86 (35.02, 67.15)	46.94 (33.17, 65.22)	-0.04 (-0.08, -0.01)	49.47 (35.43, 68.6)	72.52 (51.14, 101.53)	0.47 (0.42, 0.51)	0.33 (0.01, 0.65)
Middle SDI	845843600.95 (819383997.55, 870422101.71)	1215345443.16 (1168552422.1, 1261131506.92)	505653.92 (359587.99, 703114.26)	812668.96 (572963.7, 1126099.74)	0.61 (0.54, 0.68)	59.78 (42.51, 83.13)	66.87 (47.14, 92.66)	0.12 (0.07, 0.17)	48.52 (34.54, 67.44)	77.38 (54.44, 107.63)	0.59 (0.53, 0.66)	0.24 (-0.16, 0.64)

Notes: Age-standardized deaths rate is computed by direct standardization with global standard population in GBD 2021. Net drifts are estimates derived from the age-period-cohort model and denotes overall annual percentage change in deaths, which captures the contribution of the effects from calendar time and successive birth cohorts. Parentheses for all GBD health estimate indicate 95% uncertainty intervals; parentheses for net drift indicate 95% confidence intervals. SDI = Socio-demographic Index; APC = age-period-cohort; GBD = The Global Burden of Disease study

Regionally, the highest PCOS AAIR in 2021 was observed in high SDI regions at 90.13 (95% CI: 66.94 to 122.36) per 100,000 individuals, while low SDI regions had the lowest AAIR of 36.8 (95% CI: 25.83 to 52.19) per 100,000. High SDI regions also exhibited the highest ASIR of 144.89 (95% CI: 107.28 to 196.81) per 100,000, compared to the lowest ASIR of 27.79 (95% CI: 19.81 to 38.87) per 100,000 in low SDI regions. Projections from the APC model showed divergent net drift trends between these regions.

At the national level, the highest numbers of PCOS cases in 2021 were reported in India (346,723.54, 95% CI: 248,263.77 to 476,794.28), China (258,930.01, 95% CI: 183,411.57 to 361,501.06), the USA (194,555.53, 95% CI: 142,842.67 to 258,038.33), Indonesia (143,576.89, 95% CI: 102,491.88 to 199,866.65), and Mexico (89,661.69, 95% CI: 63,147.57 to 124,689.16). New Zealand reported the highest AAIR at 169.33 (95% CI: 120.33 to 233.47) per 100,000, while Bosnia and Herzegovina had the lowest at 4.6 (95% CI: 3.12 to 6.67) per 100,000. Italy had the highest ASIR at 326.18 (95% CI: 227.34 to 458.58) per 100,000, while Bosnia and Herzegovina reported the lowest at 7.59 (95% CI: 5.07 to 11.17) per 100,000. Net drift varied, with Georgia showing an increase of 0.71 (95% CI: -1.12 to 2.56) and Poland showing a decrease of -2.24 (95% CI: -2.93 to -1.54) (Fig. 2, Supplementary Tables 1 and Supplementary Fig. 1).

### Temporal trends in PCOS incidence across age groups

**Fig. 3:**
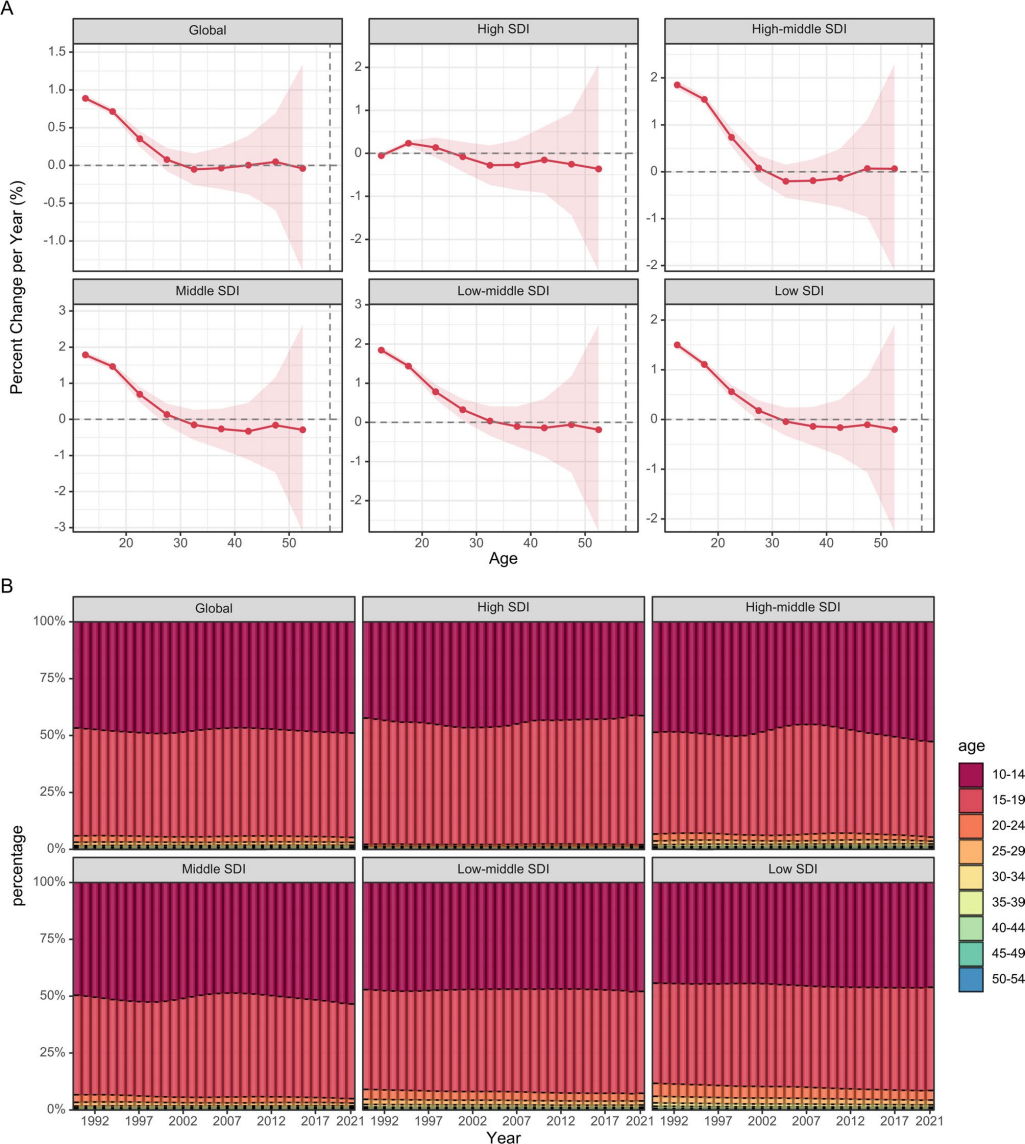
Local drifts of incidence rate and age distribution of incidences by SDI quintiles. 1990–2021. (**A**) Local drifts of PCOS incidence rate (estimates from age-period-cohort models) for 9 age groups (10–14, 15–19, 20–24, …, 50–54 years), 1990–2021. The dots and shaded areas indicate the annual percentage change of incidence rate (% per year) and the corresponding 95% CI. (**B**) Temporal change in the relative proportion of PCOS incidences across 9 age groups (10–14, 15–19, 20–24, 25–29, …, 45–49, 50–54 years), 1990–2021. PCOS = Polycystic ovary syndrome; SDI = Socio-demographic Index. CI = Confidence interval

Figure 3A illustrates a global rise in PCOS incidence across most age groups between 1990 and 2021. The 10–14 age group exhibited the highest annual growth rate of 0.887% (95% CI: 0.843–0.932%), while the annual growth rate generally decreased with age. The 30–34 age group experienced the smallest decrease in growth rate at -0.053% (95% CI: -0.260–0.156%). This pattern was consistent across most regions, except for high SDI regions, where incidence rates declined in all age groups, excluding 15–19 and 20–24 years. The 50–54 age group in high SDI regions showed the most substantial annual decrease of -0.364% (95% CI: -2.739–2.069%), highlighting significant regional differences.

Figure 3B presents changes in the age distribution of PCOS incidence over time. In 1990, 90% of global PCOS cases were found in individuals under 20 years old, with the 10–14 and 15–19 age groups each accounting for nearly half of the cases. This distribution remained largely unchanged through 2021. A similar pattern was observed across most regions, except for high SDI regions, where PCOS cases were predominantly concentrated in the 10–14 and 15–19 age groups, with little incidence in older age groups. Further details on age-specific PCOS incidence in different countries can be found in Supplementary Fig. 2.

### Effects of age, period, and cohort on PCOS incidence rates

**Fig. 4:**
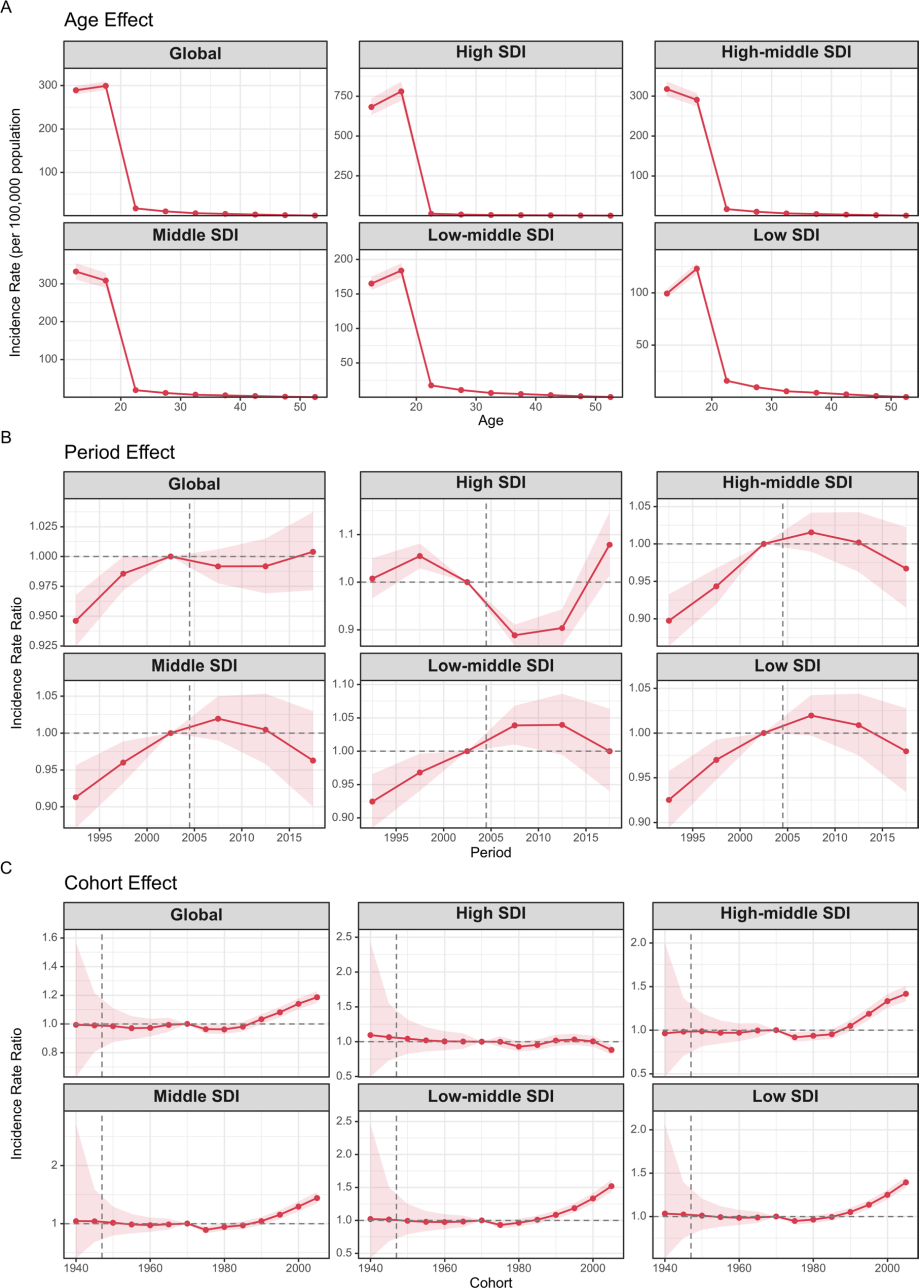
Age, period and cohort effects on PCOS incidence by SDI quintiles. (**A**) Age effects are shown by the fitted longitudinal age curves of incidence (per 100,000 person-years) adjusted for period deviations. (**B**) Period effects are shown by the relative risk of incidence (incidence rate ratio) and computed as the ratio of age-specific rates from 1990 to 1994 (the referent period 2000 to 2004) to 2015 to 2019. (**C**) Cohort effects are shown by the relative risk of incidence and computed as the ratio of age-specific rates from the 1940 cohort to the 2005 cohort, with the referent cohort set at 1970. The dots and shaded areas denote incidence rates or rate ratios and their corresponding 95% CI. PCOS = Polycystic ovary syndrome; SDI = Socio-demographic Index. CI = Confidence interval

Figure 4 explores the impact of age, period, and cohort on PCOS incidence, categorized by SDI quintile. Age-related trends were consistent across all SDI quintiles, with the highest incidence observed in the 10–14 and 15–19 age groups, while incidence remained low in older age groups. In high, low-middle, and low SDI regions, the 15–19 age group had the highest incidence, while in high-middle and middle SDI regions, the 10–14 age group exhibited the highest incidence (Fig. 4A).

The period effect showed a global increase in PCOS incidence over time (Fig. 4B). Most SDI regions followed a similar pattern of rising risk, which peaked around 2005 before declining. However, high SDI regions experienced a decline in risk from 1997 to 2013, followed by a sharp increase after 2013.

The cohort effect revealed a steady rise in PCOS risk for birth cohorts born after 1985, reaching a peak in the 2005 cohort. This trend was consistent across high-middle, middle, low-middle, and low SDI regions. In contrast, high SDI regions exhibited relatively stable risk across cohorts, with the 2005 cohort showing the lowest incidence risk among all birth cohorts (Fig. 4C). Further details on the age, period, and cohort effects across various countries are provided in Supplementary Figs. 3–5.

## Discussion

### Global and regional incidence patterns

The analysis revealed a substantial increase in PCOS cases, rising from approximately 1,476,225 in 1990 to 2,301,505 in 2021, with an age-standardized incidence rate of 63.26 per 100,000 individuals by 2021. Regionally, high SDI regions reported the highest ASIR at 144.89 per 100,000, contrasting sharply with low SDI regions at 27.79 per 100,000. This disparity is exemplified by Italy, which recorded the highest national ASIR at 326.18 per 100,000. This exceptionally high rate likely reflects a combination of factors: early and widespread adoption of the broader Rotterdam diagnostic criteria—known to increase case ascertainment by over two-fold relative to NIH criteria heightened clinical awareness and surveillance among adolescent gynecologists [23, 24] and an appreciable burden of metabolic risk factors in youth (21.2% adult obesity prevalence) [25]. Moreover, emerging evidence implicates environmental endocrine‐disrupting chemicals in altering ovarian folliculogenesis in prepubertal populations, which may contribute disproportionately to elevated incidence in the 10–14 age group [26]. Conversely, countries like Bosnia and Herzegovina, with a rate of 7.59 per 100,000, may underreport due to limited access to specialized gynecological services, as noted in studies on diagnostic delays in resource-limited settings [27].

At the national level, the highest numbers of cases were reported in India (346,723.54), China (258,930.01), the USA (194,555.53), Indonesia (143,576.89), and Mexico (89,661.69), reflecting population size and varying diagnostic capacities. New Zealand, with an ASIR of 236.5 per 100,000, highlights the influence of high SDI status on reported rates, potentially driven by better healthcare infrastructure. This contrasts with countries like Mexico and Ecuador, where despite high obesity rates (a major PCOS risk factor) [28, 29] fragmented healthcare systems delay diagnosis until fertility complications arise [30]. These disparities underscore the interplay between SDI, diagnostic capacity, and true epidemiological burden.

### Factors influencing PCOS incidence

The APC analysis disentangled the effects of age, period, and cohort on PCOS incidence. Age-related trends showed the highest incidence in the 10–14 and 15–19 age groups, accounting for nearly 90% of cases in 1990 and 2021, aligning with the biological onset of symptoms at menarche [31]. This pattern was consistent across most regions, except in high SDI areas where incidence declined in older age groups (e.g., 50–54 years, with an annual decrease of -0.364%), possibly due to effective lifestyle interventions targeting older women [32].

Period effects indicated a global rise in incidence risk peaking around 2005, followed by a decline, which may mirror global obesity trends that escalated in the late 20th century before stabilizing in some regions [33]. High SDI regions, however, experienced a sharp increase post-2013, potentially linked to the adoption of the Rotterdam diagnostic criteria, which include milder phenotypes and thus inflate reported cases [34] or advancements in ultrasound technology [35].

Cohort effects revealed a steady rise in PCOS risk for birth cohorts born after 1985, peaking in the 2005 cohort, particularly in high-middle, middle, low-middle, and low SDI regions. This trend may reflect increasing exposure to environmental factors, such as endocrine-disrupting chemicals like bisphenol A, linked to hormonal imbalances [36]. For instance, in India, rapid urbanization and dietary shifts toward processed foods may amplify this cohort risk, contributing to 346,723 cases in 2021 [37]. In contrast, high SDI regions exhibited relatively stable risk across cohorts, with the 2005 cohort showing the lowest incidence risk, possibly due to earlier preventive measures or differing reproductive patterns, such as delayed childbearing [38].

### Interventions and public health strategies

Addressing the rising incidence of PCOS requires targeted interventions, particularly given the concentration of cases in younger age groups. Early screening programs are essential, especially in low SDI regions where adolescent healthcare is often inadequate [27]. Mobile ultrasound units or telemedicine, as piloted in rural settings for other health conditions, could improve diagnostic access in these areas. In high SDI regions, where incidence rates are high, focusing on lifestyle interventions such as weight management programs has been shown to reduce risk in older women, with studies demonstrating effectiveness in reducing PCOS symptoms [32].

Public health strategies should also address modifiable risk factors, including obesity, sedentary behavior, and exposure to endocrine-disrupting chemicals. For example, promoting physical activity and healthy diets, successful in high SDI settings, could be adapted for middle and low SDI regions undergoing rapid economic transitions, such as Indonesia with 143,576 cases in 2021.

### Socioeconomic inequalities and regional disparities

The variation in incidence rates across SDI regions underscores significant health inequalities. High SDI regions report higher incidence rates, which may reflect both better diagnostic practices and higher actual incidence due to lifestyle factors such as obesity and sedentary behavior [39]. In contrast, low SDI regions may have lower reported rates due to limited healthcare infrastructure, potentially underestimating the true burden of PCOS. This inequality highlights the need for equitable access to healthcare and standardized diagnostic criteria, ensuring that resource-limited areas like Bosnia and Herzegovina can improve detection rates. The higher incidence in high SDI regions, despite declining trends in older age groups, suggests that historical diagnostic advancements and lifestyle factors associated with industrialization, such as increased obesity rates, may contribute to sustained high rates.

### Management strategies by socioeconomic context

Tailored strategies are required based on a country’s socioeconomic conditions. High SDI regions should prioritize lifestyle interventions, such as weight management programs, to reduce risk in all age groups [32]. These regions can also invest in research to understand and mitigate the effects of population aging on PCOS incidence, providing models for developing countries. Low and middle SDI regions should focus on enhancing diagnostic access, improving health education, and implementing preventive measures to address the rising cohort risk. Globally, efforts to reduce exposure to endocrine-disrupting chemicals and promote healthy dietary and physical activity habits are crucial in curbing the incidence of PCOS. Additionally, making full use of existing resources, such as mobile health units, could play an indispensable role in dealing with high disease burden in these countries [40].

### Study limitations and strengths

This study is based on data from the GBD 2021 database, which may have variations in data collection methods across countries, leading to potential biases. Underdiagnosis in low SDI regions is a notable limitation, potentially resulting in underestimated incidence rates. However, the use of standardized modeling techniques in the GBD methodology, such as network meta-regression and spatiotemporal Gaussian process regression, helps mitigate some of these issues. While earlier GBD-based studies established foundational trends in PCOS burden, this study advances the field by quantifying the independent effects of age, period, and cohort on incidence—a methodological innovation critical for designing temporally targeted interventions. This comprehensive approach strengthens our understanding of global PCOS trends and informs the development of targeted public health interventions. However, it is worth noting that this study’s reliance on the SDI, while effective for capturing health-related socioeconomic disparities, may not fully account for broader socioeconomic factors such as education and income. The Human Development Index (HDI), which integrates life expectancy, education, and per capita income, could serve as a valuable complementary metric in future research.

### Future research directions

To build upon the findings of this study, future research should prioritize addressing the diagnostic disparities observed, particularly in low SDI regions where underdiagnosis is prevalent. Investigating innovative diagnostic tools or methods that can be implemented in resource-limited settings could significantly improve detection rates. Additionally, studies evaluating the effectiveness of lifestyle interventions tailored to different socioeconomic contexts are needed to inform public health strategies. Exploring the role of specific environmental factors, such as endocrine-disrupting chemicals, in the pathogenesis of PCOS could provide insights into preventive measures. Furthermore, research on the long-term health outcomes of PCOS, including its impact on aging populations, is crucial for understanding the full burden of the disease. Finally, evaluating the impact of public health policies and interventions on PCOS incidence could guide future efforts to mitigate the global burden of this condition.

## Conclusion

This APC analysis refines our understanding of PCOS incidence trends, revealing nuanced drivers obscured in earlier prevalence-focused studies. By contextualizing rising case numbers within diagnostic evolution, and generational environmental exposures, this work provides a framework for stratified interventions. Future efforts must harmonize diagnostic criteria globally while addressing modifiable risks—particularly in transitioning economies where cohort effects are most pronounced.

## Electronic supplementary material

Below is the link to the electronic supplementary material.

Supplementary Material 1

Supplementary Material 2

## Data Availability

No datasets were generated or analysed during the current study.
