# Supplementary material for: Global trends in polycystic ovarian syndrome over 30 years: an age-period-cohort study of 204 countries and territories (1990–2021)
Source: J Health Popul Nutr. 2025 Jul 8;44:242. doi: 10.1186/s41043-025-01002-1 (PMC12239280; doi:10.1186/s41043-025-01002-1)
Supplement: Supplementary file 2 — Supplementary Material 2 [file 41043_2025_1002_MOESM2_ESM.pdf]

Figure legends for supplementary figures

Figure S1. local drifts of PCOS incidence in All SDI Countries, 1990-2021.
(FS1A) local drifts of PCOS incidence in High SDI Countries, 1990-2021.
(FS1B) local drifts of PCOS incidence in High-middle SDI Countries, 1990-2021.
(FS1C) local drifts of PCOS incidence in Middle SDI Countries, 1990-2021.
(FS1D) local drifts of PCOS incidence in Low-middle SDI Countries, 1990-2021.
(FS1E) local drifts of PCOS incidence in Low SDI Countries, 1990-2021.
The dots and shaded areas indicate the values of local drift (annual percentage change in incidence) and its 95% UI for PCOS in 9 age groups (10~14 to 50-54 years).
SDI=Socio-demographic Index. PCOS= Polycystic ovary syndrome.

Figure S2. Age distribution of incidence from PCOS in All SDI countries, 1990-2021.
(FS2A) Age distribution of PCOS incidence in High SDI Countries, 1990-2021.
(FS2B) Age distribution of PCOS incidence in High-middle SDI Countries, 1990-2021.
(FS2C) Age distribution of PCOS incidence in Middle SDI Countries, 1990-2021.
(FS2D) Age distribution of PCOS incidence in Low-middle SDI Countries, 1990-2021.
(FS2E) Age distribution of PCOS incidence in Low SDI Countries, 1990-2021.
Age distribution of incidence is represented as temporal change in the relative proportion of incidence across age groups (10-14, 15-19, 20-24, 25-29, ..., 45-49, 50-54 years) during 1990-2021.
SDI=Socio-demographic Index. PCOS= Polycystic ovary syndrome.

Figure S3. Age effects on PCOS incidence in All SDI countries.
(FS3A) Age effects of PCOS incidence in High SDI Countries.
(FS3B) Age effects of PCOS incidence in High-middle SDI Countries.
(FS3C) Age effects of PCOS incidence in Middle SDI Countries.
(FS3D) Age effects of PCOS incidence in Low-middle SDI Countries.
(FS3E) Age effects of PCOS incidence in Low SDI Countries.
Age effects indicate age-associated natural history and are shown by the fitted longitudinal age curves of incidence (per 100000 person-years) adjusted for period deviations, with the dots denoting incidence rates with 95% UI.
SDI=Socio-demographic Index. PCOS= Polycystic ovary syndrome.

Figure S4. Period effects on PCOS incidence in All SDI countries.
(FS4A) Period effects of PCOS incidence in High SDI Countries.
(FS4B) Period effects of PCOS incidence in High-middle SDI Countries.
(FS4C) Period effects of PCOS incidence in Middle SDI Countries.
(FS4D) Period effects of PCOS incidence in Low-middle SDI Countries.
(FS4E) Period effects of PCOS incidence in Low SDI Countries.
Period effects are shown by the relative risk of incidence (incidence rate ratio) for each period from 1990-1994 to 2015-2019, with the dots and shaded areas representing rate ratios and 95% UI for a given period relative to the referent period (2000-2004).
SDI=Socio-demographic Index. PCOS= Polycystic ovary syndrome.

Figure S5. Cohort effects on PCOS incidence in All SDI countries.
(FS5A) Cohort effects of PCOS incidence in High SDI Countries.
(FS5B) Cohort effects of PCOS incidence in High-middle SDI Countries.
(FS5C) Cohort effects of PCOS incidence in Middle SDI Countries.
(FS5D) Cohort effects of PCOS incidence in Low-middle SDI Countries.
(FS5E) Cohort effects of PCOS incidence in Low SDI Countries.
Cohort effects are shown by the relative risk of incidence (incidence rate ratio) for each birth cohort from 1940 to 2005, with the dots and shaded areas represent rate ratios and 95% UI for a given cohort relative to the referent 1970 cohort.
SDI=Socio-demographic Index. PCOS= Polycystic ovary syndrome.

Figure S1

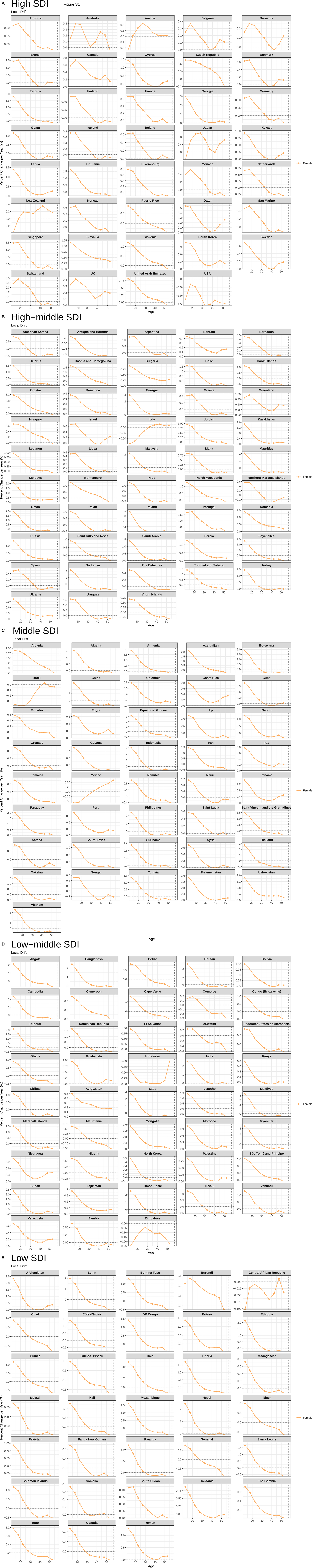
A
High SDI
Local Drift
Andorra
Australia
Austria
Belgium
Bermuda
Brunei
Canada
Cyprus
Czech Republic
Denmark
Estonia
Finland
France
Georgia
Germany
Guam
Iceland
Ireland
Japan
Kuwait
Latvia
Lithuania
Luxembourg
Monaco
Netherlands
New Zealand
Norway
Puerto Rico
Qatar
San Marino
Singapore
Slovakia
Slovenia
South Korea
Sweden
Switzerland
UK
United Arab Emirates
USA
Percent Change per Year (%)
Age
Female
B
High−middle SDI
Local Drift
American Samoa
Antigua and Barbuda
Argentina
Bahrain
Barbados
Belarus
Bosnia and Herzegovina
Bulgaria
Chile
Cook Islands
Croatia
Dominica
Georgia
Greece
Greenland
Hungary
Israel
Italy
Jordan
Kazakhstan
Lebanon
Libya
Malaysia
Malta
Mauritius
Moldova
Montenegro
Niue
North Macedonia
Northern Mariana Islands
Oman
Palau
Poland
Portugal
Romania
Russia
Saint Kitts and Nevis
Saudi Arabia
Serbia
Seychelles
Spain
Sri Lanka
The Bahamas
Trinidad and Tobago
Turkey
Ukraine
Uruguay
Virgin Islands
Percent Change per Year (%)
Age
Female
C
Middle SDI
Local Drift
Albania
Algeria
Armenia
Azerbaijan
Botswana
Brazil
China
Colombia
Costa Rica
Cuba
Ecuador
Egypt
Equatorial Guinea
Fiji
Gabon
Grenada
Guyana
Indonesia
Iran
Iraq
Jamaica
Mexico
Namibia
Nauru
Panama
Paraguay
Peru
Philippines
Saint Lucia
Saint Vincent and the Grenadines
Samoa
South Africa
Suriname
Syria
Thailand
Tokelau
Tonga
Tunisia
Turkmenistan
Uzbekistan
Vietnam
Percent Change per Year (%)
Age
Female
D
Low−middle SDI
Local Drift
Angola
Bangladesh
Belize
Bhutan
Bolivia
Cambodia
Cameroon
Cape Verde
Comoros
Congo (Brazzaville)
Djibouti
Dominican Republic
El Salvador
eSwatini
Federated States of Micronesia
Ghana
Guatemala
Honduras
India
Kenya
Kiribati
Kyrgyzstan
Laos
Lesotho
Maldives
Marshall Islands
Mauritania
Mongolia
Morocco
Myanmar
Nicaragua
Nigeria
North Korea
Palestine
São Tomé and Príncipe
Sudan
Tajikistan
Timor−Leste
Tuvalu
Vanuatu
Venezuela
Zambia
Zimbabwe
Percent Change per Year (%)
Age
Female
E
Low SDI
Local Drift
Afghanistan
Benin
Burkina Faso
Burundi
Central African Republic
Chad
Côte d'Ivoire
DR Congo
Eritrea
Ethiopia
Guinea
Guinea−Bissau
Haiti
Liberia
Madagascar
Malawi
Mali
Mozambique
Nepal
Niger
Pakistan
Papua New Guinea
Rwanda
Senegal
Sierra Leone
Solomon Islands
Somalia
South Sudan
Tanzania
The Gambia
Togo
Uganda
Yemen
Percent Change per Year (%)
Age
Female

Figure S2

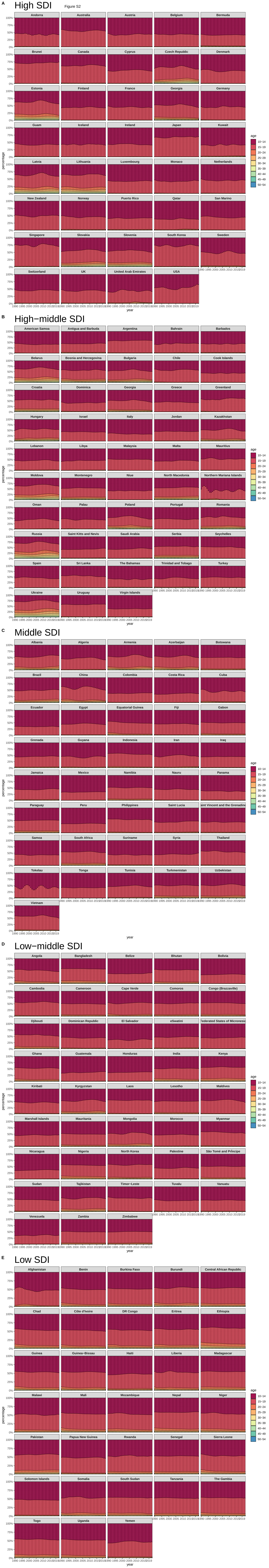
A
High SDI
Andorra
Australia
Austria
Belgium
Bermuda
Brunei
Canada
Cyprus
Czech Republic
Denmark
Estonia
Finland
France
Georgia
Germany
Guam
Iceland
Ireland
Japan
Kuwait
Latvia
Lithuania
Luxembourg
Monaco
Netherlands
New Zealand
Norway
Puerto Rico
Qatar
San Marino
Singapore
Slovakia
Slovenia
South Korea
Sweden
Switzerland
UK
United Arab Emirates
USA
B
High−middle SDI
American Samoa
Antigua and Barbuda
Argentina
Bahrain
Barbados
Belarus
Bosnia and Herzegovina
Bulgaria
Chile
Cook Islands
Croatia
Dominica
Georgia
Greece
Greenland
Hungary
Israel
Italy
Jordan
Kazakhstan
Lebanon
Libya
Malaysia
Malta
Mauritius
Moldova
Montenegro
Niue
North Macedonia
Northern Mariana Islands
Oman
Palau
Poland
Portugal
Romania
Russia
Saint Kitts and Nevis
Saudi Arabia
Serbia
Seychelles
Spain
Sri Lanka
The Bahamas
Trinidad and Tobago
Turkey
Ukraine
Uruguay
Virgin Islands
C
Middle SDI
Albania
Algeria
Armenia
Azerbaijan
Botswana
Brazil
China
Colombia
Costa Rica
Cuba
Ecuador
Egypt
Equatorial Guinea
Fiji
Gabon
Grenada
Guyana
Indonesia
Iran
Iraq
Jamaica
Mexico
Namibia
Nauru
Panama
Paraguay
Peru
Philippines
Saint Lucia
Saint Vincent and the Grenadines
Samoa
South Africa
Suriname
Syria
Thailand
Tokelau
Tonga
Tunisia
Turkmenistan
Uzbekistan
Vietnam
D
Low−middle SDI
Angola
Bangladesh
Belize
Bhutan
Bolivia
Cambodia
Cameroon
Cape Verde
Comoros
Congo (Brazzaville)
Djibouti
Dominican Republic
El Salvador
eSwatini
Federated States of Micronesia
Ghana
Guatemala
Honduras
India
Kenya
Kiribati
Kyrgyzstan
Laos
Lesotho
Maldives
Marshall Islands
Mauritania
Mongolia
Morocco
Myanmar
Nicaragua
Nigeria
North Korea
Palestine
São Tomé and Príncipe
Sudan
Tajikistan
Timor−Leste
Tuvalu
Vanuatu
Venezuela
Zambia
Zimbabwe
E
Low SDI
Afghanistan
Benin
Burkina Faso
Burundi
Central African Republic
Chad
Côte d'Ivoire
DR Congo
Eritrea
Ethiopia
Guinea
Guinea−Bissau
Haiti
Liberia
Madagascar
Malawi
Mali
Mozambique
Nepal
Niger
Pakistan
Papua New Guinea
Rwanda
Senegal
Sierra Leone
Solomon Islands
Somalia
South Sudan
Tanzania
The Gambia
Togo
Uganda
Yemen
percentage
year
age
10–14
15–19
20–24
25–29
30–34
35–39
40–44
45–49
50–54

Figure S3

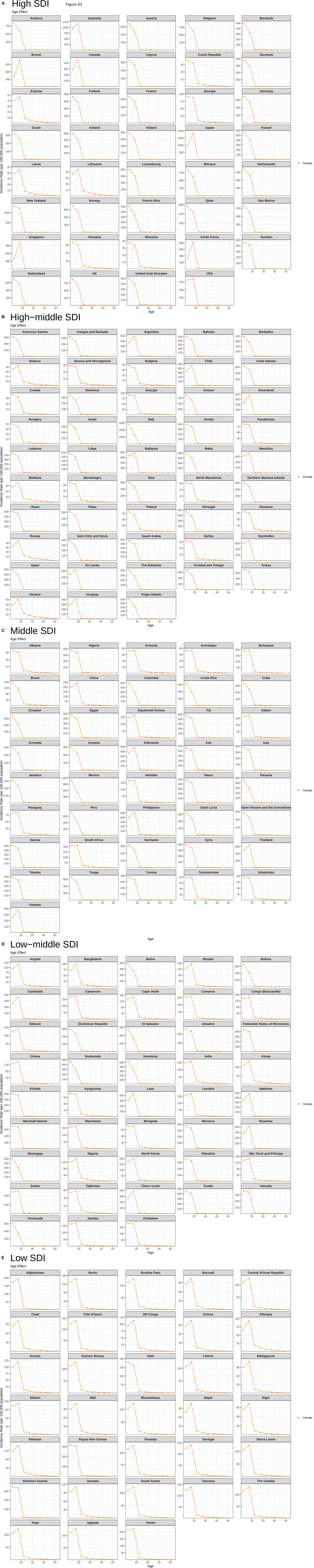
A High SDI
Age Effect
Andorra
Australia
Austria
Belgium
Bermuda
Brunei
Canada
Cyprus
Czech Republic
Denmark
Estonia
Finland
France
Georgia
Germany
Guam
Iceland
Ireland
Japan
Kuwait
Latvia
Lithuania
Luxembourg
Monaco
Netherlands
New Zealand
Norway
Puerto Rico
Qatar
San Marino
Singapore
Slovakia
Slovenia
South Korea
Sweden
Switzerland
UK
United Arab Emirates
USA
Incidence Rate (per 100,000 population)
Age
Female
B High−middle SDI
Age Effect
American Samoa
Antigua and Barbuda
Argentina
Bahrain
Barbados
Belarus
Bosnia and Herzegovina
Bulgaria
Chile
Cook Islands
Croatia
Dominica
Georgia
Greece
Greenland
Hungary
Israel
Italy
Jordan
Kazakhstan
Lebanon
Libya
Malaysia
Malta
Mauritius
Moldova
Montenegro
Niue
North Macedonia
Northern Mariana Islands
Oman
Palau
Poland
Portugal
Romania
Russia
Saint Kitts and Nevis
Saudi Arabia
Serbia
Seychelles
Spain
Sri Lanka
The Bahamas
Trinidad and Tobago
Turkey
Ukraine
Uruguay
Virgin Islands
Incidence Rate (per 100,000 population)
Age
Female
C Middle SDI
Age Effect
Albania
Algeria
Armenia
Azerbaijan
Botswana
Brazil
China
Colombia
Costa Rica
Cuba
Ecuador
Egypt
Equatorial Guinea
Fiji
Gabon
Grenada
Guyana
Indonesia
Iran
Iraq
Jamaica
Mexico
Namibia
Nauru
Panama
Paraguay
Peru
Philippines
Saint Lucia
Saint Vincent and the Grenadines
Samoa
South Africa
Suriname
Syria
Thailand
Tokelau
Tonga
Tunisia
Turkmenistan
Uzbekistan
Vietnam
Incidence Rate (per 100,000 population)
Age
Female
D Low−middle SDI
Age Effect
Angola
Bangladesh
Belize
Bhutan
Bolivia
Cambodia
Cameroon
Cape Verde
Comoros
Congo (Brazzaville)
Djibouti
Dominican Republic
El Salvador
eSwatini
Federated States of Micronesia
Ghana
Guatemala
Honduras
India
Kenya
Kiribati
Kyrgyzstan
Laos
Lesotho
Maldives
Marshall Islands
Mauritania
Mongolia
Morocco
Myanmar
Nicaragua
Nigeria
North Korea
Palestine
São Tomé and Príncipe
Sudan
Tajikistan
Timor−Leste
Tuvalu
Vanuatu
Venezuela
Zambia
Zimbabwe
Incidence Rate (per 100,000 population)
Age
Female
E Low SDI
Age Effect
Afghanistan
Benin
Burkina Faso
Burundi
Central African Republic
Chad
Côte d'Ivoire
DR Congo
Eritrea
Ethiopia
Guinea
Guinea−Bissau
Haiti
Liberia
Madagascar
Malawi
Mali
Mozambique
Nepal
Niger
Pakistan
Papua New Guinea
Rwanda
Senegal
Sierra Leone
Solomon Islands
Somalia
South Sudan
Tanzania
The Gambia
Togo
Uganda
Yemen
Incidence Rate (per 100,000 population)
Age
Female

Figure S4

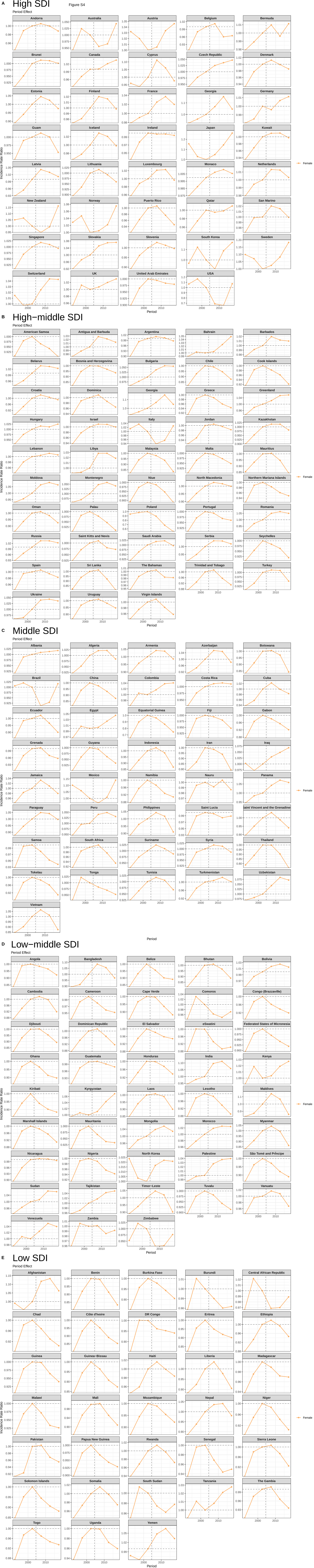
A High SDI
Period Effect
Andorra
Australia
Austria
Belgium
Bermuda
Brunei
Canada
Cyprus
Czech Republic
Denmark
Estonia
Finland
France
Georgia
Germany
Guam
Iceland
Ireland
Japan
Kuwait
Latvia
Lithuania
Luxembourg
Monaco
Netherlands
New Zealand
Norway
Puerto Rico
Qatar
San Marino
Singapore
Slovakia
Slovenia
South Korea
Sweden
Switzerland
UK
United Arab Emirates
USA
Incidence Rate Ratio
Period
Female
B High−middle SDI
Period Effect
American Samoa
Antigua and Barbuda
Argentina
Bahrain
Barbados
Belarus
Bosnia and Herzegovina
Bulgaria
Chile
Cook Islands
Croatia
Dominica
Georgia
Greece
Greenland
Hungary
Israel
Italy
Jordan
Kazakhstan
Lebanon
Libya
Malaysia
Malta
Mauritius
Moldova
Montenegro
Niue
North Macedonia
Northern Mariana Islands
Oman
Palau
Poland
Portugal
Romania
Russia
Saint Kitts and Nevis
Saudi Arabia
Serbia
Seychelles
Spain
Sri Lanka
The Bahamas
Trinidad and Tobago
Turkey
Ukraine
Uruguay
Virgin Islands
Incidence Rate Ratio
Period
Female
C Middle SDI
Period Effect
Albania
Algeria
Armenia
Azerbaijan
Botswana
Brazil
China
Colombia
Costa Rica
Cuba
Ecuador
Egypt
Equatorial Guinea
Fiji
Gabon
Grenada
Guyana
Indonesia
Iran
Iraq
Jamaica
Mexico
Namibia
Nauru
Panama
Paraguay
Peru
Philippines
Saint Lucia
Saint Vincent and the Grenadines
Samoa
South Africa
Suriname
Syria
Thailand
Tokelau
Tonga
Tunisia
Turkmenistan
Uzbekistan
Vietnam
Incidence Rate Ratio
Period
Female
D Low−middle SDI
Period Effect
Angola
Bangladesh
Belize
Bhutan
Bolivia
Cambodia
Cameroon
Cape Verde
Comoros
Congo (Brazzaville)
Djibouti
Dominican Republic
El Salvador
eSwatini
Federated States of Micronesia
Ghana
Guatemala
Honduras
India
Kenya
Kiribati
Kyrgyzstan
Laos
Lesotho
Maldives
Marshall Islands
Mauritania
Mongolia
Morocco
Myanmar
Nicaragua
Nigeria
North Korea
Palestine
São Tomé and Príncipe
Sudan
Tajikistan
Timor−Leste
Tuvalu
Vanuatu
Venezuela
Zambia
Zimbabwe
Incidence Rate Ratio
Period
Female
E Low SDI
Period Effect
Afghanistan
Benin
Burkina Faso
Burundi
Central African Republic
Chad
Côte d'Ivoire
DR Congo
Eritrea
Ethiopia
Guinea
Guinea−Bissau
Haiti
Liberia
Madagascar
Malawi
Mali
Mozambique
Nepal
Niger
Pakistan
Papua New Guinea
Rwanda
Senegal
Sierra Leone
Solomon Islands
Somalia
South Sudan
Tanzania
The Gambia
Togo
Uganda
Yemen
Incidence Rate Ratio
Period
Female

Figure S5

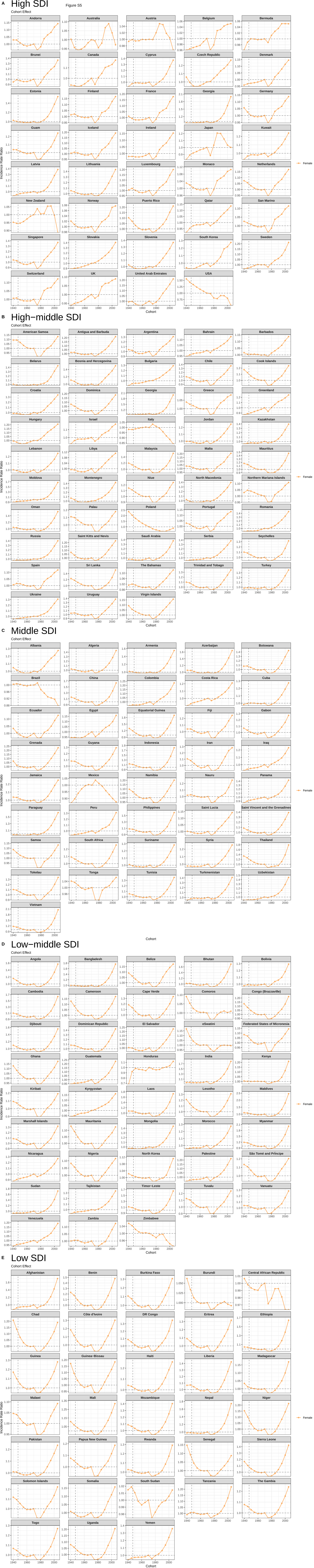

A High SDI
Cohort Effect
Andorra
Australia
Austria
Belgium
Bermuda
Brunei
Canada
Cyprus
Czech Republic
Denmark
Estonia
Finland
France
Georgia
Germany
Guam
Iceland
Ireland
Japan
Kuwait
Latvia
Lithuania
Luxembourg
Monaco
Netherlands
New Zealand
Norway
Puerto Rico
Qatar
San Marino
Singapore
Slovakia
Slovenia
South Korea
Sweden
Switzerland
UK
United Arab Emirates
USA
Incidence Rate Ratio
Cohort
Female
B High−middle SDI
Cohort Effect
American Samoa
Antigua and Barbuda
Argentina
Bahrain
Barbados
Belarus
Bosnia and Herzegovina
Bulgaria
Chile
Cook Islands
Croatia
Dominica
Georgia
Greece
Greenland
Hungary
Israel
Italy
Jordan
Kazakhstan
Lebanon
Libya
Malaysia
Malta
Mauritius
Moldova
Montenegro
Niue
North Macedonia
Northern Mariana Islands
Oman
Palau
Poland
Portugal
Romania
Russia
Saint Kitts and Nevis
Saudi Arabia
Serbia
Seychelles
Spain
Sri Lanka
The Bahamas
Trinidad and Tobago
Turkey
Ukraine
Uruguay
Virgin Islands
Incidence Rate Ratio
Cohort
Female
C Middle SDI
Cohort Effect
Albania
Algeria
Armenia
Azerbaijan
Botswana
Brazil
China
Colombia
Costa Rica
Cuba
Ecuador
Egypt
Equatorial Guinea
Fiji
Gabon
Grenada
Guyana
Indonesia
Iran
Iraq
Jamaica
Mexico
Namibia
Nauru
Panama
Paraguay
Peru
Philippines
Saint Lucia
Saint Vincent and the Grenadines
Samoa
South Africa
Suriname
Syria
Thailand
Tokelau
Tonga
Tunisia
Turkmenistan
Uzbekistan
Vietnam
Incidence Rate Ratio
Cohort
Female
D Low−middle SDI
Cohort Effect
Angola
Bangladesh
Belize
Bhutan
Bolivia
Cambodia
Cameroon
Cape Verde
Comoros
Congo (Brazzaville)
Djibouti
Dominican Republic
El Salvador
eSwatini
Federated States of Micronesia
Ghana
Guatemala
Honduras
India
Kenya
Kiribati
Kyrgyzstan
Laos
Lesotho
Maldives
Marshall Islands
Mauritania
Mongolia
Morocco
Myanmar
Nicaragua
Nigeria
North Korea
Palestine
São Tomé and Príncipe
Sudan
Tajikistan
Timor−Leste
Tuvalu
Vanuatu
Venezuela
Zambia
Zimbabwe
Incidence Rate Ratio
Cohort
Female
E Low SDI
Cohort Effect
Afghanistan
Benin
Burkina Faso
Burundi
Central African Republic
Chad
Côte d'Ivoire
DR Congo
Eritrea
Ethiopia
Guinea
Guinea−Bissau
Haiti
Liberia
Madagascar
Malawi
Mali
Mozambique
Nepal
Niger
Pakistan
Papua New Guinea
Rwanda
Senegal
Sierra Leone
Solomon Islands
Somalia
South Sudan
Tanzania
The Gambia
Togo
Uganda
Yemen
Incidence Rate Ratio
Cohort
Female
